# *In vivo* characterization of the novel ebolavirus Bombali virus suggests a low pathogenic potential for humans

**DOI:** 10.1080/22221751.2022.2164216

**Published:** 2023-01-18

**Authors:** B. S. Bodmer, A. Breithaupt, M. Heung, J. E. Brunetti, C. Henkel, J. Müller-Guhl, E. Rodríguez, L. Wendt, S. L. Winter, M. Vallbracht, A. Müller, S. Römer, P. Chlanda, C. Muñoz-Fontela, T. Hoenen, B. Escudero-Pérez

**Affiliations:** a Institute for Molecular Virology and Cell Biology, Friedrich-Loeffler-Institut, Greifswald – Insel Riems, Germany; b Department of Experimental Animal Facilities and Biorisk Management, Friedrich-Loeffler-Institut, Greifswald – Insel Riems, Germany; c Bernhard Nocht Institute for Tropical Medicine, Hamburg, Germany; d Leibniz Institute of Virology, Hamburg, Germany; e German Center for Infection Research (DZIF), Partner Site Hamburg-Luebeck-Borstel, Braunschweig, Germany; f Schaller Research Groups, Department of Infectious Diseases, Virology, Heidelberg University Hospital, Heidelberg, Germany

**Keywords:** Bombali virus, ebolavirus, filovirus, virulence, pathogenic potential, reverse genetics, full-length clone, humanized mice

## Abstract

Ebolaviruses cause outbreaks of haemorrhagic fever in Central and West Africa. Some members of this genus such as Ebola virus (EBOV) are highly pathogenic, with case fatality rates of up to 90%, whereas others such as Reston virus (RESTV) are apathogenic for humans. Bombali virus (BOMV) is a novel ebolavirus for which complete genome sequences were recently found in free-tailed bats, although no infectious virus could be isolated. Its pathogenic potential for humans is unknown. To address this question, we first determined whether proteins encoded by the available BOMV sequence found in *Chaerephon pumilus* were functional in *in vitro* assays. The correction of an apparent sequencing error in the glycoprotein based on these data then allowed us to generate infectious BOMV using reverse genetics and characterize its infection of human cells. Furthermore, we used HLA-A2-transgenic, NOD-scid-IL-2γ receptor-knockout (NSG-A2) mice reconstituted with human haematopoiesis as a model to evaluate the pathogenicity of BOMV *in vivo* in a human-like immune environment. These data demonstrate that not only does BOMV show a slower growth rate than EBOV *in vitro*, but it also shows low pathogenicity in humanized mice, comparable to previous studies using RESTV. Taken together, these findings suggest a low pathogenic potential of BOMV for humans.

## Introduction

The genus *Ebolavirus* comprises six virus species, all of which are classified as risk group 4 agents [1]. The most prominent member is Ebola virus (EBOV; species *Zaire ebolavirus*), which has been responsible for numerous Ebola virus disease (EVD) outbreaks in Central and West Africa, including the largest one to date with more than 28,500 cases and at least 11,000 deaths [2]. However, not all ebolaviruses cause disease, and in particular Reston virus (RESTV; species *Reston ebolavirus*) appears apathogenic, despite multiple documented infections [3]. Recently, genome sequences for a novel ebolavirus, Bombali virus (BOMV; species *Bombali ebolavirus*) were identified in free-tailed bats (*Mops condylurus* and *Chaerephon pumilus*) in Sierra Leone [4], and later in Guinea and Kenya [5, 6], suggesting that this virus is present throughout large areas of sub-Saharan Africa. However, to date no human cases of BOMV infection have been reported, and thus the pathogenic potential of this virus and its risk to public health remain unknown. This issue is further exacerbated by the fact that, until now, no infectious BOMV isolate has been available. However, it has previously been shown that a recombinant vesicular stomatitis virus (VSV) encoding BOMV GP can enter human cells, suggesting the possibility of BOMV infecting human cells [4]. Nevertheless, further studies are required to determine the risk that this novel filovirus may pose for humans.

Animal models are commonly used to investigate the pathogenesis of ebolaviruses. Unfortunately, with the exception of non-human primate models, they either rely on virus-adaptation to the model animal species, do not authentically reflect differences in the pathogenic potential of different filoviruses for humans, and/or do not recapitulate hallmarks of pathogenesis in humans such as systemic cytokine production, coagulopathy, and liver damage [7]. To address this issue, we have recently established a humanized mouse model using HLA-A2-transgenic NOD-scid-IL-2γ receptor-knockout (huNSG-A2) mice reconstituted with human haematopoiesis, which not only show these three key hallmarks of pathogenesis, but also recapitulate the case-fatality rates of different ebolaviruses in humans without the need for virus adaptation to the animals [8]. Specifically, while the human-pathogenic EBOV causes 90%−100% lethality in this model, the human-apathogenic RESTV killed only 20% of infected mice. We, thus, sought to use this model to experimentally assess the pathogenic potential of BOMV.

Reverse genetics systems, and specifically full-length clone systems, allow the generation of infectious ebolaviruses from cDNA (reviewed in [9]). These systems are based on the generation of a viral antigenomic cRNA and its replication into genomic vRNA for subsequent transcription by the ebolavirus nucleoprotein NP, the polymerase L, the polymerase cofactor VP35, and the transcriptional activator VP30 [10], which together with the vRNA also form the ribonucleoprotein complex (RNP). This results in the production of all viral proteins, including also the viral matrix protein VP40 (responsible for morphogenesis and budding) [11, 12], the viral glycoprotein GP (responsible for entry into target cells) [13], and the nucleocapsid-associated protein VP24 (responsible for generating packaging-competent nucleocapsids containing the RNP proteins and genomic vRNA) [14], thus initiating the virus life cycle. In the past, full-length clone systems were generated based on sequences from existing virus isolates; however, by using *de novo* gene synthesis it is also possible to generate viruses for which no isolates exist, such as BOMV.

A second application of reverse genetics is the generation of life cycle-modelling systems, which use minigenomes to model aspects of the virus life cycle (reviewed in [15]). Classical minigenome systems use a minigenome composed of only a single easily assayable reporter gene flanked by authentic filovirus genome ends, which when coexpressed together with the RNP proteins results in viral replication and transcription of the minigenome, leading to reporter activity reflecting these processes [10]. As an extension of this system, the viral genes encoding VP40, GP and VP24 can also be included in the minigenome. This results in the expression of these additional proteins and allows formation of transcription and replication-competent virus-like particles (trVLPs), which package minigenomes and can infect target cells. Consequently, such a tetracistronic trVLP system models almost all aspects of the virus life cycle [14].

In this study, we generated infectious BOMV *de novo* using reverse genetics after confirming the functionality of its individual genes using life cycle-modelling systems, and correcting a single mistake in the published sequence that rendered the glycoprotein non-functional. We then characterized this virus in tissue culture as well as our humanized mouse model. Our results indicate that BOMV behaves similarly to RESTV, which we have previously extensively characterized in humanized mice [8], and in particular shows a similar low pathogenic potential in this model, suggesting that BOMV is likely also apathogenic for humans.

## Materials and methods

### Cells

VeroE6 and Huh7 (kindly provided by Stephan Becker, Philipps Universität-Marburg), HEK293T (Collection of Cell Lines in Veterinary Medicine CCLV-RIE1018) and A549 (CCLV-RIE1035) cells were maintained in Dulbecco’s Modified Eagle’s medium (DMEM) supplemented with 100 U/mL penicillin and 100 µg/mL streptomycin and 1x GlutaMAX (all Thermo Fisher Scientific) and fetal bovine serum (10% for maintenance or 5% for experiments) at 37°C with 5% CO_2_.

### VLP and trVLP assay

For the VLP assay, flag-tagged EBOV or BOMV VP40 was cloned into pCAGGS and expressed in HEK293T cells. Amounts of VP40 in cell lysates and supernatants were assessed 48 h post-transfection by Western blotting using an anti-flag antibody. For trVLP assays, heterologous exchange of the BOMV GP gene into the EBOV tetracistronic minigenome was performed using a type IIS restriction enzyme-based approach. Otherwise trVLP assays were performed as previously described [14]. For further details see Supplemental Methods.

### Rescue, titration and growth kinetics of recombinant virus

The recombinant EBOV used in this study has been previously described [16]. To generate recombinant BOMV, a modified version of the full-length sequence isolated from *C. pumilus* (GenBank accession number MF319186) but incorporating silent mutations to facilitate cloning (see Supplemental Methods), as well as the non-synonymous mutation c7702t (GP.P554L), was synthesized and cloned into pAmp [17]. All other plasmids for rescue have been previously described [14, 18] and the rescue was performed as previously described for EBOV [19]. For further details see Supplemental Methods. The sequence of the rescued virus was confirmed by Sanger sequencing and submitted to GenBank (Accession number ON871047). Virus titration was performed by tissue culture infectious dose 50 (TCID_50_) assay, with incubation of the samples for 27 days to ensure clear CPE development for BOMV. Growth kinetics were performed in 12-well plates on VeroE6 and A549 cells using a multiplicity of infection of 0.01 as previously described [20].

### Cryo-electron tomography

For cryo-electron tomography, virus (inactivated by 4% paraformaldehyde and 0.1% glutaraldehyde prior to removal from BSL4) or VLP samples were mixed with colloidal gold, vitrified, and data were obtained on a Titan Krios Transmission Electron Microscope (ThermoFisher Scientific). Tilt series were reconstructed using the IMOD software package [21]. For further details see Supplemental Methods.

### Generation and infection of humanized mice

Generation of humanized mice was performed as previously described [8]. Briefly, 5-week-old NSG-A2 (HLA-A2.1) females were sublethally irradiated (240 cGy). Four hours after irradiation, mice underwent intravenous (retro-orbital) transplantation of 1 × 10^6^ HLA-A2–matching CD34^+^ haematopoietic stem cells per mouse, which had been positively selected from cord blood. Eight weeks after transplantation, blood samples were collected, and the presence of human haematopoietic cells was quantified by flow cytometry. All infection experiments were performed at week 10 after engraftment. Animals were intranasally infected with 1 × 10^5^ TCID_50_ of recombinant EBOV or BOMV, or mock-infected with DMEM. Mice were monitored daily, and those showing weight loss of more than 20% of their original body weight were euthanized according to the approved study protocol.

### Immunofocus assay

Virus titres in blood and organ samples were quantified by immunofocus assay as previously described, but with slight modifications [8]. Briefly, dilution series of homogenized samples were incubated on VeroE6 cells with a 1% methylcellulose overlay, and fixed for immunostaining using an anti-NP antibody 7 days after infection. For further details see Supplemental Methods.

### Histopathology

Tissue samples were collected, fixed in 10% neutral-buffered formalin and trimmed for paraffin embedding, and 2–3 μm-thick sections were stained with haematoxylin and eosin (HE). Consecutive slides were processed for immunohistochemistry (IHC) using an anti-NP antibody. Slides were scanned using a Hamamatsu S60 scanner, and evaluation and interpretation were performed by a board-certified pathologist using the post-examination masking method [22]. For details see Supplemental Methods.

Of note, it was not always possible to distinguish between apoptosis and single-cell necrosis based on HE staining. For better readability, and because EBOV is known to primarily induce apoptosis, the term “apoptosis” is used. Due to the low number of animals, statistical analysis was not performed for evaluation of histopathologic changes. Graphical illustrations were prepared using GraphPad Prism v9.0.0 (GraphPad Software). Dots represent individual animals scores, and lines indicate the median for ordinal scores.

### Approvals

Human hematopoietic stem cells were isolated from cord blood obtained at the Asklepios Klinik Nord in Hamburg with informed written consent by all patients and under a protocol approved by the Ethics Commission of the Medical Association of Hamburg (WF-054/15). Animal experiments were performed under a study protocol approved by the German animal protection authorities (Behörde für Gesundheit und Verbraucherschutz, Hamburg, approval 110/17). Animals were housed in individually ventilated cages. All work with infectious EBOV or BOMV was carried out in BSL4 laboratories of the Bernhard Nocht Institute and the Friedrich-Loeffler-Institut following approved standard operating procedures.

### Dual use statement

Work on BOMV described in this manuscript was assessed by the Biorisk Committee of the Friedrich-Loeffler-Institut for potential dual use issues, and no such issues were identified.

## Results

### Functional validation of published Bombali virus sequences

**Figure 1.**
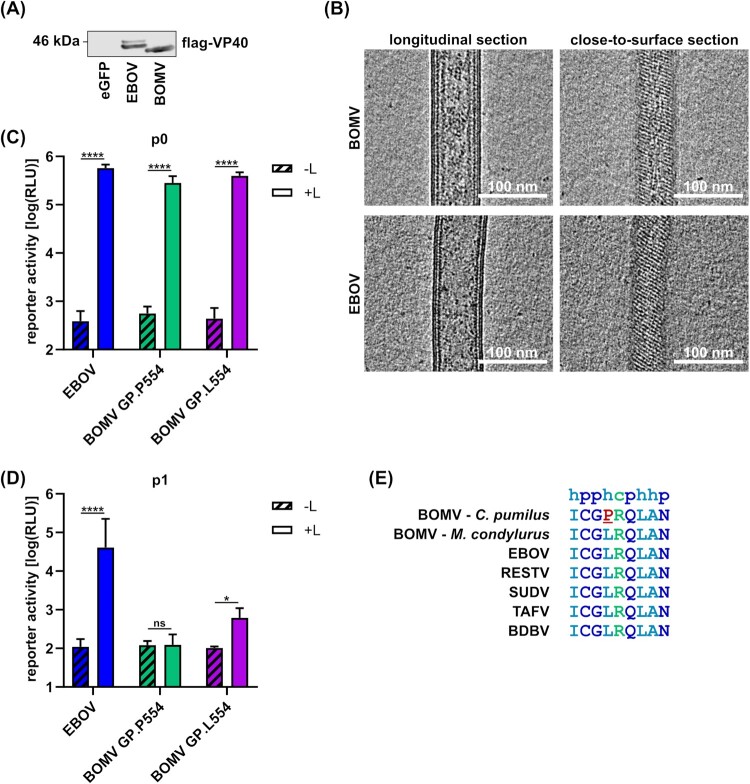
Functional characterization of BOMV proteins. (A) VLP formation induced by BOMV VP40. HEK293T cells were transfected with expression plasmids for flag-tagged EBOV or BOMV VP40 (or eGFP as a negative control). Supernatants were harvested 48 h post-transfection and analyzed via SDS-PAGE and Western blot. VP40 was detected with an anti-flag antibody. (B) Cryo-electron tomography of VLPs composed of BOMV VP40 or EBOV VP40. Longitudinal and near-to-surface slices through a tomogram of VLPs are shown. The VP40 matrix forms helically ordered linear segments underneath the inner membrane monolayer that are visible as regular striations from the top view. Scale bars: 100 nm. (C)-(D) Functionality of BOMV GP. HEK293T cells (p0 cells) were transfected with all components for a trVLP assay, including EBOV tetracistronic minigenome plasmids encoding EBOV or BOMV GP either as published for *C. pumilus* (GP.P554), or with a single proline to leucine exchange at amino acid position 554 (GP.L554). As a negative control the expression plasmid for L was omitted (-L). Reporter activity in p0 cells (C) was measured 72 h post-transfection, and trVLPs were harvested and used to infect p1 cells, in which reporter activity was measured 72 h post-infection (D). Means and standard deviation of 4 biological replicates from 2 independent experiments are shown. Significant differences compared to -L controls are indicated: n.s.: not significant; *: *p* < 0.5; ****: *p* < 0.0001. (E) Comparison of the amino acid sequences of the heptad repeat 1 region of GP2 from different ebolaviruses. h = hydrophobic, *p* = polar, c = charged. The helix-breaking proline residue specific to the published BOMV sequence from *C. pumilus* (MF319186) is shown in red and underlined.

As a first step towards generating BOMV by means of reverse genetics we sought to functionally validate the BOMV sequence found in *C. pumilus*. We had previously shown that this sequence encodes functional RNP proteins, and that its terminal leader and trailer regions contain functional replication and transcription promoters [23]. To assess whether the VP40 gene also encodes a functional protein, we assessed the ability of BOMV VP40 to facilitate the production of VLPs, which is characteristic for ebolavirus matrix proteins [24, 25]. As expected, BOMV VP40 was able to induce VLP formation to a similar extent than EBOV VP40 (Figure 1(A)). These VLPs were investigated by cryo-electron tomography and showed an identical morphology to VLPs produced by EBOV VP40, including the typical regular VP40 striations seen near the surface of particles that is consistent with VP40 organization found in infectious EBOV [12, 26] (Figure 1(B)).

To assess the sequence of the GP gene, we exchanged the EBOV GP gene in an existing tetracistronic EBOV minigenome against that of BOMV GP. While BOMV GP did not affect reporter-activity in trVLP-producing cells (Figure 1(C)), we observed a complete loss of trVLP infectivity (Figure 1(D)). This was surprising because the closely related BOMV sequence found in *M. condylurus* has been shown to encode a functional GP [4]. We, therefore, compared the BOMV GP sequences found in *C. pumilus* and *M. condylurus* with other ebolaviruses (Supplemental Figure 1). This analysis showed seven amino acids that were different in the *C. pumilus* BOMV GP sequence compared to all other ebolavirus GP sequences. Five of these were at positions with no or little sequence conservation and one was a conservative change from a glutamic acid to an aspartic acid. However, the remaining difference entailed a leucine to proline exchange at a highly conserved position involved in forming an α-helix that is crucial for fusion (Supplemental Figure 1, Figure 1(E)) [27]. Introduction of a single nucleotide substitution reverted the encoded amino acid from the helix-breaking proline to the highly conserved leucine, and restored glycoprotein function (Figure 1(D)). We subsequently used this modified sequence for our attempts to rescue infectious BOMV.

### Rescue and in vitro characterization of recombinant Bombali virus

**Figure 2.**
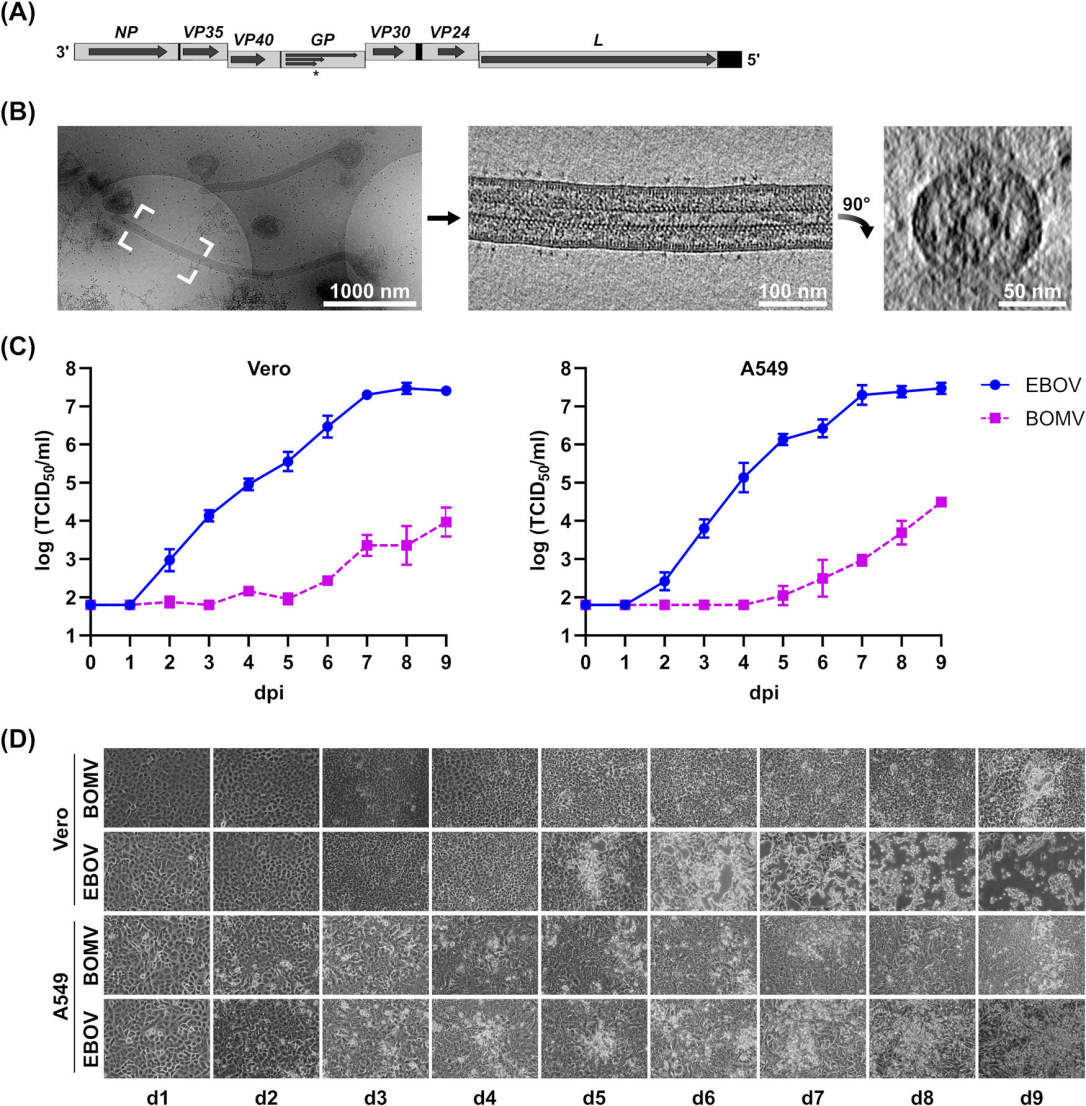
*In vitro* characterization of recombinant BOMV. (A) Genome structure of recombinant BOMV. The full-length genome is shown, with genes depicted as grey boxes and open reading frames indicated as arrows. Black boxes show non-transcribed regions, and steps indicate gene overlaps. The asterisk marks the editing site in the GP gene giving rise to sGP, GP_1,2_ and ssGP. (B) Cryo-electron tomography of recombinant BOMV. An overview 2D projection image of BOMV distributed on an electron microscopy grid is shown, along with slices of a tomogram showing the longitudinal cross-section of the filamentous Bombali virus highlighted, and a transverse cross-section. Scale bars: 1 µm (overview), 100 nm (longitudinal cross-section), and 50 nm (transverse cross-section). (C) Growth kinetics of EBOV and BOMV. VeroE6 or A549 cells were infected with recombinant EBOV (circles) or BOMV (squares) at a multiplicity of infection of 0.01 and supernatants were harvested daily. Viral titres were analyzed by tissue culture infectious dose 50 (TCID_50_) assay. dpi = days post-infection. (D) Development of cytopathic effect. Infected cells from (C) were examined daily for the development of cytopathic effect. d = day.

Rescue of recombinant BOMV (Figure 2(A)) resulted in cytopathic effect (CPE) about 4 weeks after blind passage, which is much later than during rescue of other filoviruses. Nonetheless, harvested virus stocks had a titre of 2 × 10^6^ TCID_50_/ml, comparable with the titres of recombinant EBOV stocks. Sanger sequencing of the harvested virus showed no mutations. The morphology of BOMV virions was examined using cryo-electron tomography, and showed characteristic filamentous particles with a diameter of 94 nm (Figure 2(B)). For *in vitro* characterization, we compared the growth of BOMV and EBOV in VeroE6 and A549 cells (Figure 2(C)). In both cell lines, BOMV showed significantly reduced growth (Figure 2(C)), and while EBOV-infected cells showed extensive CPE, in BOMV-infected cells only very limited CPE was observed (Figure 2(D)).

### Infection of humanized mice with recombinant Bombali virus

**Figure 3.**
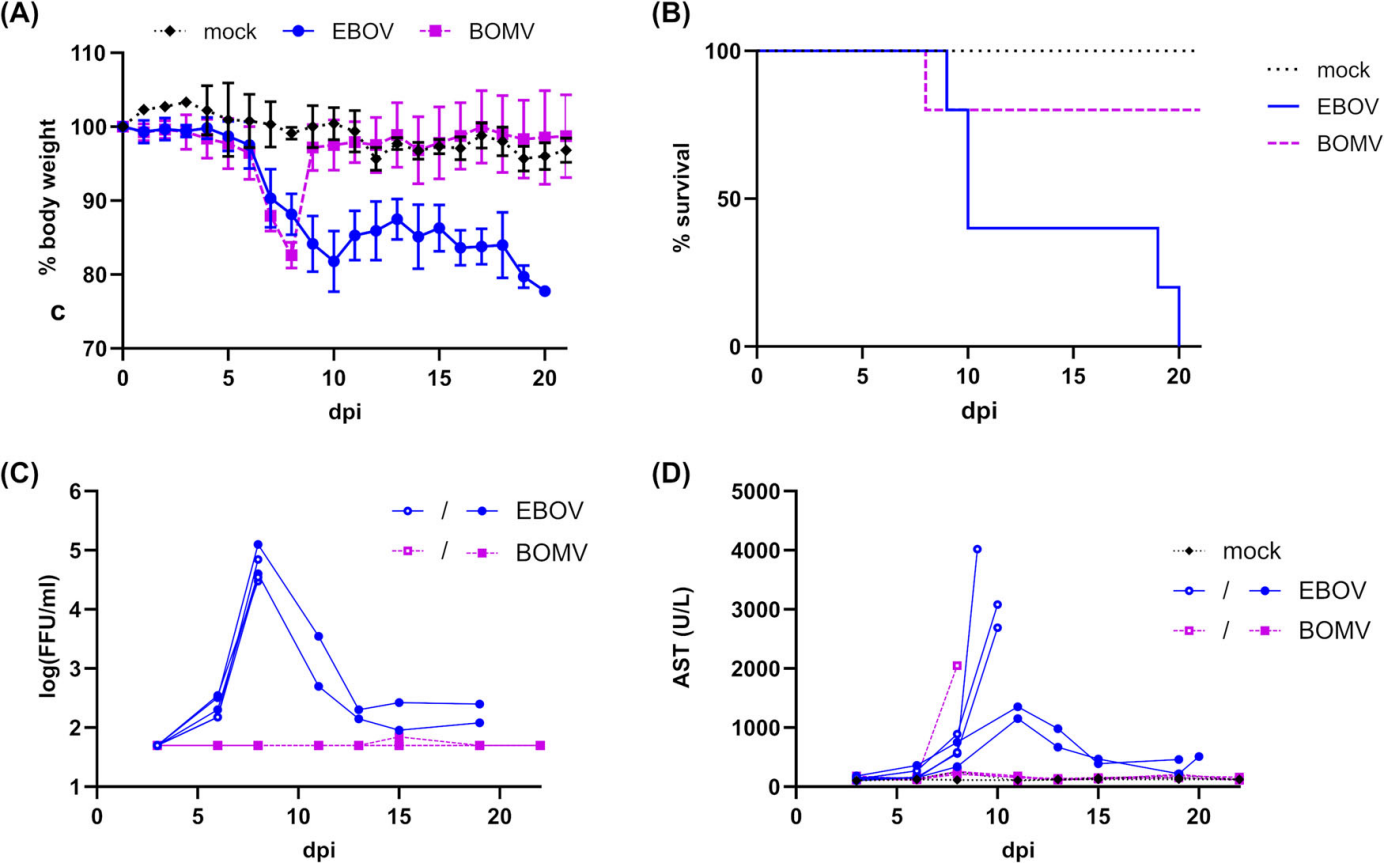
*In vivo* characterization of recombinant BOMV in infected huNSG-A2 mice. (A) Weight loss in EBOV and BOMV infected mice. Mice were infected intranasally with EBOV (*n* = 5) or BOMV (*n* = 5), whereas mock-infected mice (*n* = 3) received 20 μL DMEM. Means and standard deviations for each group are shown. A nonparametric Mann-Whitney test indicated significant differences between results for mice infected with BOMV and EBOV (*p* = 0.0147). dpi = days post-infection. (B) Kaplan-Meier survival curves of EBOV and BOMV infected mice. A log-rank (Mantel-Cox) test indicated statistically significant differences between BOMV and EBOV-infected mice (*p* = 0.0350). (C) Viremia and (D) AST levels in the blood of EBOV and BOMV surviving and non-surviving mice. Individual animals are shown, with non-survivors and survivors indicated as empty circles/squares or filled circles/squares, respectively. dpi = days post-infection.

To assess the pathogenic potential of BOMV, we utilized the huNSG-A2 humanized mouse model [8]. Importantly, prior to infection, the engraftment of human hematopoietic cells in peripheral blood in huNSG-A2 mice was comparable in the different experimental groups (Supplemental Figure 2). All mice infected with either EBOV or BOMV showed signs of morbidity (weight loss) starting around day 6 after inoculation (Figure 3(A)). However, only one of the BOMV-infected animals met the humane endpoint and had to be euthanized at day 8. Thus, the overall fatality rate in our model was 20%, identical to what we have previously observed for RESTV infection [8]. In contrast, infection with EBOV was uniformly lethal (Figure 3(B)). Interestingly, the EBOV-infected mice fell into two groups, with the first 3 mice dying at day 9 or day 10, whereas the remaining mice showed a prolonged disease course. This is in line with previous results in this model, where death after EBOV infection occurred between day 9 and 20 post-infection [8].

Levels of viremia and serum aminotransferases (AST) are good predictors of the outcome in humans as well as in animal models [8, 28, 29, 30]. We observed that all surviving BOMV-infected mice showed undetectable levels of viremia (Figure 3(C)) and maintained very low levels of serum AST (Figure 3(D)). This is similar to what we have previously observed in surviving RESTV-infected animals, which showed very low virus titres and no increase in AST [8]. In contrast, the BOMV-infected animal that died after infection showed high levels of AST immediately prior to death, even though no viremia could be detected. All EBOV-infected mice showed high levels of viremia at day 8; however, in the mice that died late after infection, this viremia was transient. Animals that died early after infection with EBOV also showed very high AST levels immediately prior to death. In contrast, animals that died late after EBOV-infection showed only a moderate transient increase in AST levels around day 11 post-infection, and no pronounced increase in AST levels occurred prior to death. For the limited number of RESTV-infected mice succumbing to infection we have previously observed a pattern resembling that of EBOV-infected animals dying late in this study, with a transient AST increase between days 11 and 15 post-infection, and a transient peak in viremia at the same time [8].

**Figure 4.**
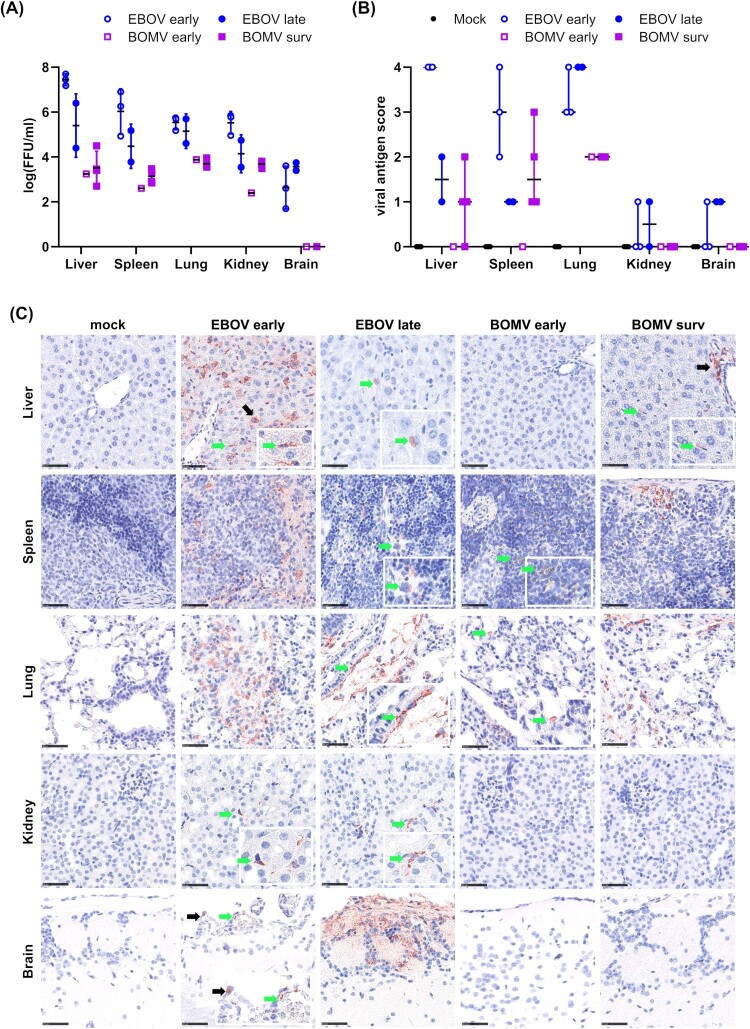
Organ virus and antigen loads in infected huNSG-A2 mice. (A) Viral titres in organs. VeroE6 cells were inoculated with homogenized organ samples from EBOV- or BOMV-infected huNSG-A2 mice, as indicated. Infected cells were overlaid with methylcellulose containing medium for 7 days. After fixation and permeabilization, infection foci were visualized and counted. FFU = focus forming units. (B) Immunohistochemistry of EBOV and BOMV infected mice. Fixed tissue samples from the indicated organs were sliced, processed for immunohistochemistry, and evaluated for viral antigen scoring (see Supplemental Table 1). Scores for individual animals as well as median scores are shown, with non-survivors and survivors indicated as empty circles/squares or filled circles/squares, respectively. (C) Representative tissue slices used for the evaluation of viral antigen scores. Arrows highlight viral antigen (red-brown signal), and insets show a magnification of the area. EBOV antigen was found in the liver in both hepatocytes (black arrow) and sinusoid lining cells (green arrow); in the spleen in mononuclear immune cells, where it was widespread (EBOV early) or found in scattered cells (EBOV late, green arrow); in the lung where it was abundant in interstitial mononuclear cells (EBOV early) and in perivascular interstitial mesenchymal cells (EBOV late, green arrow); in the kidney in a few stellate cells in the interstitium (green arrow); in the brain in meningeal mesenchymal cells (EBOV early, black arrow) and endothelial cells (EBOV early, green arrow) and as widespread labelling in olfactory nerve cells (EBOV late). BOMV antigen was found in the liver in periportal mesenchymal cells (BOMV survivor, black arrow) and sinusoid lining cells (green arrow); in the spleen in mononuclear cells, in single cells (BOMV early, green arrow) and subcapsular (BOMV survivor); in the lung in alveolar cells that could not be not further characterized (BOMV early, green arrow) and in interstitial immune cells or mesenchymal cells (BOMV survivor). No BOMV antigen was found in the kidney or in the brain. Scale bars indicate 50 µm.

**Figure 5.**
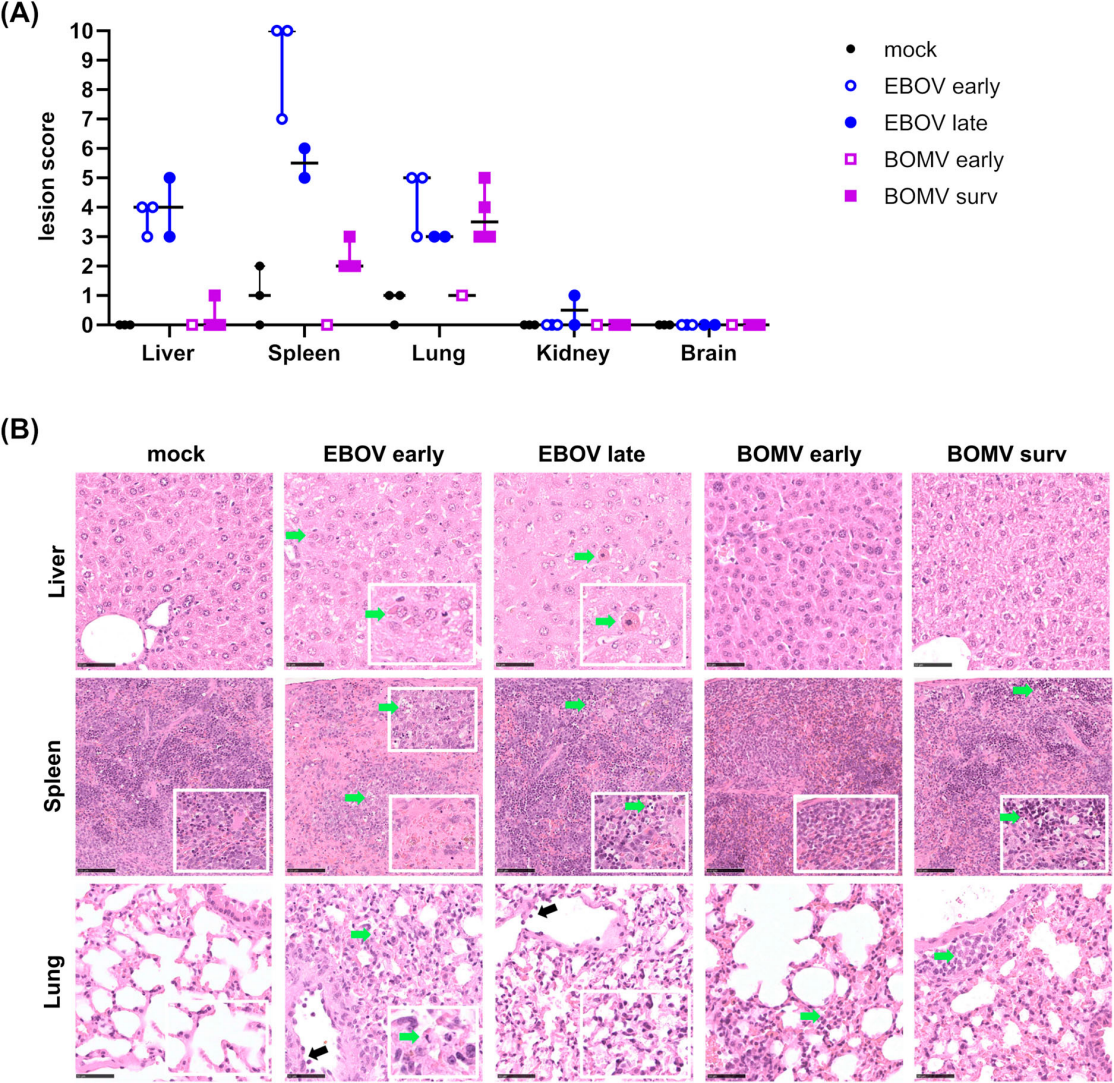
Histopathologic lesions in organs of EBOV and BOMV infected huNSG-A2 mice. (A) Lesion scores from tissue slices. Slices from the indicated organs of EBOV- or BOMV-infected huNSG-A2 mice were stained with hematoxylin and eosin and evaluated for histopathological changes (see Supplemental Table 2). Values for individual animals as well as median scores are shown, with non-survivors and survivors indicated as empty circles/squares or filled circles/squares, respectively. (B) Representative tissue slices used for the evaluation of lesion scores. Mock-infected mice showed no changes in the liver, spleen (lower inlay showing expected cellularity for comparison) or lung; EBOV infection was associated with the presence of cytoplasmic viral inclusion bodies (EBOV early, green arrow), and Councilman bodies (EBOV late, green arrow); in the spleen, an EBOV-induced decrease in cellularity (EBOV early, lower inlay) and apoptosis of lymphocytes in the red and white pulp with numerous tingible body macrophages (EBOV early and late, green arrow) was observed. EBOV infection also affected the lung showing diffuse septal thickening, alveolar apoptosis (green arrow), activation of the endothelium with immune cell rolling (black arrow) and interstitial, mononuclear infiltrates (EBOV late, inlay). No lesions were seen in the liver after BOMV infection; in the spleen apoptosis of lymphocytes in the red pulp with numerous tingible body macrophages (BOMV late, green arrow) was seen; in the lung BOMV infection-associated interstitial (BOMV early, green arrow) and perivascular (BOMV late, green arrow) mononuclear infiltrates were observed. Scale bars in liver and lung samples indicate 50 µm, and in spleen samples 100 µm.

High virus titres in organs are also associated with severe disease in animal models [8, 31]. Titration of samples harvested at the time of death (or in surviving animals at day 21 post-infection) by immunofocus assay showed EBOV titres in all organs tested, whereas samples from BOMV-infected animals showed only low titres in spleen, liver, lung and kidney, and no detectable titres in brain samples (Figure 4(A)). These findings were also reflected in immunohistochemistry and histopathology, even though interpretation of the data requires caution given the limited number of animals, which is due to the nature of the model. Nonetheless, in the tested animals, immunohistochemistry showed that EBOV infection led to a more widespread tissue tropism and a slightly broader spectrum of target cells compared to BOMV (Figure 4(B and C), Supplemental Table 1). Earlier death (days 9–10) of EBOV-infected animals was usually associated with a more widespread antigen detection compared to later time points (days 19–20). In clear contrast, BOMV antigen was restricted to the lung on day 8 post-infection and to the liver, lung and spleen on day 21, whereas the kidney and brain were consistently negative for BOMV antigen throughout the study. Within the liver, lung and spleen, BOMV largely exhibited the same spectrum of target cells as EBOV, although antigen was less abundant (Supplemental Table 1). Similarly, in surviving RESTV-infected animals we have previously observed antigen staining in the liver to a much lesser extent than in EBOV-infected animals [8]. In contrast, antigen staining in lung and spleen was comparable between EBOV and RESTV, and staining in the kidney for RESTV-infected animals even exceeded that of EBOV-infected animals. Correlating with viral antigen detection, histopathologic findings after EBOV or BOMV infection were most evident in the liver, spleen and lung, and the total lesion scores obtained were generally higher after EBOV infection compared to BOMV infection (Figure 5(A and B)). Details for the histopathologic lesions are given in Supplemental Table 2.

## Discussion

The increasing frequency with which new viruses are being identified highlights the need to be able to rapidly assess whether such viruses could be pathogenic for humans, and thus pose a risk for future emergence. While *in vitro* studies can provide some insights [20, 32, 33, 34, 35], more complex systems such as animal models are required to ultimately assess such a risk. In this study, we have used our huNSG-A2 humanized mouse model to assess the pathogenic potential of BOMV that was generated *de novo* by reverse genetics. These animals contain mature human peripheral immune cells and have previously been shown to recapitulate EVD symptoms observed in humans without the need for virus adaptation [8]. Intranasal inoculation was used as an infection route in order to mimic the events that take place during mucosal exposure, which is one of the most common routes of infection during EVD outbreaks [36, 37]. However, this did not result in pronounced differences in disease course or outcome compared to intraperitoneal infection [38]. Further, we used an extremely robust infection dose (1 × 10^5^ TCID_50_), which was 10x higher than in our previous studies [8, 38], in order to ensure that also a limited pathogenic potential of BOMV would not be underestimated.

Indeed, while due to the very complex and resource-intense nature of our animal model animal numbers in our study were low, we nonetheless observed very clear differences between EBOV- and BOMV-infected animals. Importantly, the outcomes of BOMV infection much more closely resembled the data from RESTV-infected groups included as part of previous studies where we extensively compared pathogenesis and infection outcome between EBOV and RESTV in this model [8]. Specifically, we observed a dramatically reduced pathogenic potential for BOMV compared to EBOV, which went hand-in-hand with lower virus titres and antigen loads in analyzed tissues for BOMV, reduced histopathology, and in most cases an absence of hallmarks of fulminant EBOV infection such as inflammation or elevated AST values. However, it is worth noting that BOMV titres in organs, while remaining low, did not vanish, indicating a lack of clearance by the study end date. This is consistent with previous data using this model, where animals surviving infections with different ebolaviruses (EBOV, RESTV, Bundibugyo virus, Sudan virus, and Taï Forest virus) also showed low levels of viremia that could be detected at similarly late time points [8].

The specific reasons why EBOV and BOMV display such remarkable differences in pathogenic potential are not yet known. Importantly, however, despite these differences in pathology and clinical outcome, there was a high degree of concordance between EBOV and BOMV with respect to the type of affected target cells. This would appear to support a previous report showing infection of human cells by VSV encoding BOMV GP [4]. Further, this supports the fact that, already *in vitro*, BOMV replicates much slower than EBOV, which is again similar to the human apathogenic RESTV [17, 20, 39]. This difference may allow the host immune system enough time to mount a robust, but not detrimentally exacerbated, immune response - contrary to what takes place during EVD [40, 41]. Indeed, even though there does not seem to be an intrinsic inability to replicate in key target cells, all of our *in vivo* data also clearly indicate slower viral replication in target organs for BOMV. Again, these findings align with previous results for RESTV, which also showed reduced replication in various mouse models [17, 36, 42].

The reduced replication efficiency of BOMV both *in vitro* and *in vivo* strongly suggests that fundamental aspects of the viral life cycle differ in their efficiency between EBOV and BOMV. Indeed, for the apathogenic RESTV we have recently shown that a similar impairment in *in vitro* growth is linked to reduced efficiency of viral RNA synthesis [20]. For BOMV, however, we rather found that (even after the sequence was corrected) the BOMV glycoprotein was markedly reduced in its ability to mediate trVLP infection. Importantly, while these experiments were done in a heterologous context (i.e. in the background of EBOV trVLPs), interspecies and even intergenus incompatibility is not an issue in the case of the glycoprotein [18, 43] and, in particular, chimeric EBOVs encoding glycoproteins from other ebolavirus species, or even other filovirus genera, show very little attenuation [17,44].

Further, it is possible that there might be additional differences between EBOV and BOMV in their ability to antagonize host innate immune responses, which play an important role in controlling EBOV infection [45, 46]. Indeed, it has recently been suggested that BOMV VP24 might be impaired in its ability to antagonize the innate immune response compared to other ebolaviruses [32]. However, whether and to what extent these and other differences in the molecular biology of BOMV contribute to the reduced replication we observed *in vitro* and *in vivo* remain to be addressed in future.

Importantly, a current limitation to any study on BOMV is that it must rely on sequences reported in the literature, and mistakes in these sequences could affect the fitness of the rescued virus. Indeed, the sequence we chose as the basis for our recombinant virus isolate, based on the fact that it was confirmed using Sanger sequencing, which remains the gold standard in terms of accuracy, nonetheless contained what we consider a clear mistake. This mutation led to a completely non-functional glycoprotein, making it impossible for a virus with this sequence to replicate, and thus be found in nature. Further, it has to be noted that the current BOMV sequence obtained from bats might be the result of persistent infections of these animals, and might therefore be attenuated compared to other BOMV variants that have yet to be identified. However, only the availability of natural occurring isolates will clarify this issue, as well as circumventing concerns that, while rescued viruses stem from a single genetic clone, natural isolates contain quasispecies that might play a role in pathogenesis. Thus, in the meantime, it will be very important to compare our findings to those using recombinant BOMV based on other published full-length sequences.

In summary, we used huNSG-A2 mice to characterize the pathogenic potential of the recently discovered filovirus BOMV generated *de novo* by reverse genetics. These data reveal that upon mucosal exposure EBOV and BOMV show dramatically different pathogenic potential in these animals, and the currently available data suggest that BOMV likely has a low pathogenic potential for humans. Further studies are now required to characterize in more detail the specific mechanisms that underlie these differences.

## Supplementary Material

Supplemental_Tables.pdf

Supplemental_Figures.pdf

Supplemental_Methods_v5.pdf

## Data Availability

The authors confirm that the data supporting the findings of this study are available within the article and its supplementary materials. The sequence of the rescued Bombali virus is available in GenBank (https://www.ncbi.nlm.nih.gov/genbank) under accession number ON871047.
