# Supplementary material for: In vivo characterization of the novel ebolavirus Bombali virus suggests a low pathogenic potential for humans
Source: Emerg Microbes Infect. 2023 Jan 18;12(1):2164216. doi: 10.1080/22221751.2022.2164216 (PMC9858441; doi:10.1080/22221751.2022.2164216)
Supplement: Supplemental_Tables.pdf [file TEMI_A_2164216_SM6957.pdf]

Supplemental Table 1: Tissue and target cell tropism of Ebola or Bombali virus in a humanized mouse model. Immunohistochemistry for Ebola nucleoprotein, early after (day 8-10, grey shading), late after (day 19-20) or surviving (day 21) the infection.

	**Animal number**	**M1**	**M2**	**M3**	**M10**	**M6**	**M8**	**M7**	**M9**	**M13**	**M11**	**M12**	**M14**	**M15**
	**Virus**	**mock**	**mock**	**mock**	**EBOV**	**EBOV**	**EBOV**	**EBOV**	**EBOV**	**BOMV**	**BOMV**	**BOMV**	**BOMV**	**BOMV**
	**Day post infection**	**21**	**21**	**21**	**9**	**10**	**10**	**19**	**20**	**8**	**21**	**21**	**21**	**21**
**Tissue**	**Cell type**													
liver	hepatocyte[$]	0	0	0	3	3	3	1	0	0	0	0	0	1
	sinusoid lining cell	0	0	0	3	2	2	2	1	0	1	0	0	2
	endothelium	0	0	0	1	1	1	0	0	0	0	0	0	0
	mesenchymal cell	0	0	0	3	2	2	1	1	1	0	1	1	1
**liver antigen score[§]**		**0**	**0**	**0**	4	4	4	2	1	1	0	1	2	1
spleen	mononuclear cell R	0	0	0	3	3	3	1	0	0	1	1	1	3
	mononuclear cell W	0	0	0	3	1	1	1	1	0	1	0	1	3
	mesenchymal cell	0	0	0	1	1	1	0	0	0	1	0	0	1
**spleen antigen score[§]**		0	0	0	4	2	3	1	1	0	1	1	2	3
lung	alveolar macrophage	0	0	0	1	1	2	2	1	1	1	0	1	0
	septal mononuclear cell	0	0	0	2	3	3	3	3	1	2	3	1	2
	mesenchymal cell	0	0	0	4	2	3	4	3	1	2	2	1	2
**lung antigen score**		**0**	**0**	**0**	4	3	3	4	4	**2**	**2**	**2**	**2**	**2**
kidney	interstitial, stellate cells	0	0	0	1	0	0	2	0	0	0	0	0	0
	glomerular cells	0	0	0	0	0	0	1	0	0	0	0	0	0
**kidney antigen score**		**0**	**0**	**0**	1	0	0	1	0	0	0	0	0	0
brain	mesenchymal cell/endothelium	0	0	0	1	0	0	1	2	0	0	0	0	0
	olfactory nerve cell layer	0	0	0	0	0	0	2	2	0	0	0	0	0
**brain antigen score**		**0**	**0**	**0**	1	0	0	1	1	**0**	**0**	**0**	**0**	**0**

Abbreviation: EBOV, Ebola virus; BOMV, Bombali virus; R, red pulp; W white pulp

[$]cell type specific antigen score in the most severely affected high power field: 0 = negative, 1 = focal to oligofocal (1- 3 foci and <5% of the particular cell type affected), 2 = multifocal (>3 foci and 6-40%), 3= coalescing (41-80%), 4 = diffuse (>81%)

[§]tissue antigen score (bold) in the most severely affected high power field: 0 = negative, 1 = focal to oligofocal (1- 3 foci and <5% of the tissue affected), 2 = multifocal (>3 foci and 6-40%), 3= coalescing (41-80%), 4 = diffuse (>81%)

Supplemental Table 2: **Histopathology of Ebola or Bombali virus infection in a humanized mouse model.** Histopathologic findings with lesions scores early after (day 8-10, grey shading), late after (day 19-20) or surviving (day 21) the infection.

	**Animal number**	**M1**	**M2**	**M3**	**M10**	**M6**	**M8**	**M7**	**M9**	**M13**	**M11**	**M12**	**M14**	**M15**
	**Virus**	**mock**	**mock**	**mock**	**EBOV**	**EBOV**	**EBOV**	**EBOV**	**EBOV**	**BOMV**	**BOMV**	**BOMV**	**BOMV**	**BOMV**
	**Day post infection**	**21**	**21**	**21**	**9**	**10**	**10**	**19**	**20**	**8**	**21**	**21**	**21**	**21**
**Tissue**	**Criteria$**													
liver	degeneration / apoptosis	0	0	0	3	2	3	3	2	0	0	0	0	0
	intracytoplasmic viral inclusion bodies§	0	0	0	1	1	1	1	0	0	0	0	0	0
	endothelial activation§#	0	0	0	0	0	0	1	1	0	1	0	0	0
**liver lesion score**		**0**	**0**	**0**	**4**	**3**	**4**	**5**	**3**	**0***	**1**	**0**	**0**	**0**
spleen	lymphoid apoptosis, W with TBM	0	0	1	2	3	3	0	0	0	0	0	0	1
	lymphoid apoptosis, R with TBM	2	0	0	3	4	4	3	3	0	2	2	2	2
	cellularity decreased lymphocytes	0	0	0	R2	R3, W2	R3	W2	W3	0	0	0	0	0
**spleen lesion score**		**2**	**0**	**1**	**7**	**10**	**10**	**5**	**6**	**0**	**2**	**2**	**2**	**3**
lung	infiltrates, interstitial	0	0	0	0	1	0	0	1	1	0	0	0	1
	infiltrates, interstitial, main cell type	0	0	0	0	L	0	0	G	L	0	0	0	L
	alveoli, septal apoptosis	0	0	0	2	1	2	1	1	0	1	0	0	1
	infiltrates, perivascular	0	0	0	1	0	1	0	0	0	2	1	2	0
	septal thickening, acelluar	0	1	0	1	1	1	1	1	0	1	1	1	1
	endothelial activation§#	1	0	0	1	0	1	1	0	0	1	1	1	0
**lung lesion score**		**1**	**1**	**0**	**5**	**3**	**5**	**3**	**3**	**1**	**5**	**3**	**4**	**3**
kidney	glomerula, apoptosis	0	0	0	0	0	0	1	0	0	0	0	0	0
**kidney lesion score**		**0**	**0**	**0**	**0**	**0**	**0**	**1**	**0**	**0**	**0**	**0**	**0**	**0**
**brain lesion score**		**0**	**0**	**0**	**0**	**0**	**0**	**0**	**0**	**0**	**0**	**0**	**0**	**0**

Abbreviation: TBM, tingible body macrophages containing phagocytized, apoptotic cells; EBOV, Ebola virus; BOMV, Bombali virus; G, neutrophilic granulocyte; L, lymphocyte; R, red pulp; W white pulp; apoptosis may also include necrosis

$ if not stated otherwise: 0 = no changes, 1 = minimal or focal to oligofocal (<5% of the tissue affected), 2 = mild or multifocal (6-40%), 3= moderate or coalescing (41-80%), 4 = severe or diffuse (>80%) changes. The sum of all values resulted in the tissue specific lesion score per animal

§ graded as present = 1, absent = 0

#endothelial activation: hypertrophy of endothelial cells with / without rolling of immune cells

*this animal presented with mild, diffuse, hepatocellular atrophy (not included in lesion score)
