# Supplementary material for: In vivo characterization of the novel ebolavirus Bombali virus suggests a low pathogenic potential for humans
Source: Emerg Microbes Infect. 2023 Jan 18;12(1):2164216. doi: 10.1080/22221751.2022.2164216 (PMC9858441; doi:10.1080/22221751.2022.2164216)
Supplement: Supplemental_Figures.pdf [file TEMI_A_2164216_SM6956.pdf]

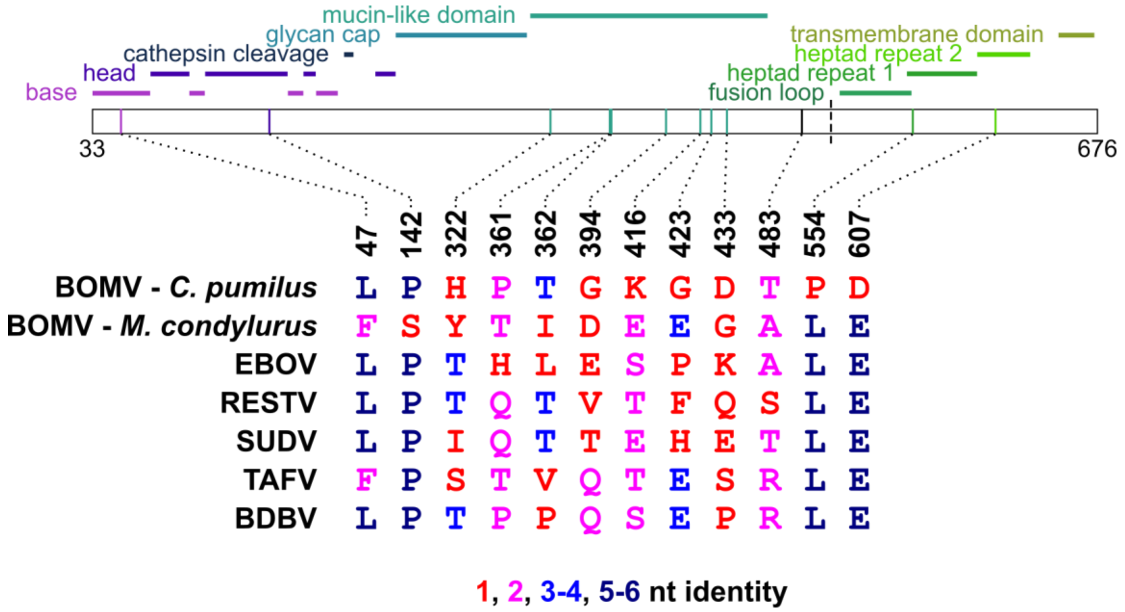

Supplemental Figure 1: Amino acid differences in GP between BOMV sequences found in *C. pumilus* and *M. condylurus*. Differences between BOMV sequences found in *C. pumilus* and *M. condylurus*, as well as the corresponding amino acids from other ebolavirus species, and their respective locations in GP are shown. Genbank IDs: BOMV - *C. pumilus*: MF319186; BOMV - *M. condylurus*: MF319185; EBOV: NC_002549; RESTV: NC_004161; SUDV: NC_006432; TAFV: NC_014372; BDBV:NC_014373.

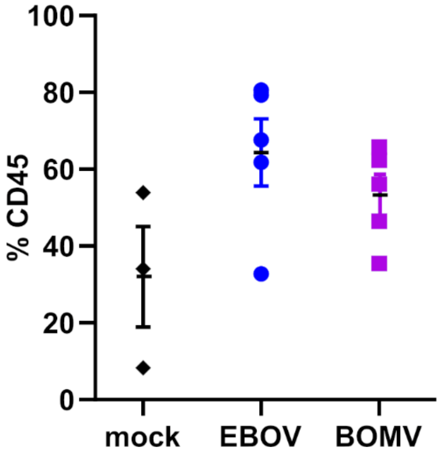

Supplemental Figure 2: Quantification of human CD45 expressing cells in EBOV and BOMV infected huNSG-A2 mice. At 12 weeks post transplantation and prior to infection, peripheral blood was analysed by flow cytometry for the presence of human hematopoietic cells using CD45 as marker. Engraftment of human cells is represented as % of $CD45^+$ cells.
