# Supplementary material for: In vivo characterization of the novel ebolavirus Bombali virus suggests a low pathogenic potential for humans
Source: Emerg Microbes Infect. 2023 Jan 18;12(1):2164216. doi: 10.1080/22221751.2022.2164216 (PMC9858441; doi:10.1080/22221751.2022.2164216)
Supplement: Supplemental_Methods_v5.pdf [file TEMI_A_2164216_SM6955.pdf]

Supplemental Methods

Cells, plasmids and viruses

For generation of the recombinant BOMV, the full-length sequence of BOMV was synthesized in 8 fragments as Gene Strings (Invitrogen), using the BOMV sequence found in *C. pumilus* (Genbank accession number MF319186) as a reference sequence, as this one has been fully Sanger sequenced. The following silent mutations were introduced in open reading frames to facilitate cloning: t1255g (dBbsI), a4031g (dXhoI), c4079t (iPvuI), t4876g (dBsmBI), a7238g (dBsmBI), g8690a (dBbsI), g9143a (dXhoI), c15595g (dNruI), t16663a (dBsmBI), c17080a (dAgeI), c17548g (dBbsI). Further, the mutation c7702t (GP.P554L) was introduced to correct a mutation in the heptad repeat 1 region of GP (see also the Results section).

The flag-tagged BOMV VP40 used in the VLP assay was cloned into pCAGGS by PCR using the primers 5'-GTT CGA ATT CAT GGA CTA CAA AGA CGA TGA CGA CAA GGG TAT GCG TAG GAC AGT GAT ACC-3' and 5'-GGT ACG CTA GCT CAT TTT TCG CTT GCG ATG G-3'.

Detailed cloning strategies are available upon request.

VLP and trVLP assays

For the VLP assay, 293T cells in 12-well format were transfected with 1000 ng pCAGGS-flag-EBOV-VP40 or pCAGGS-flag-BOMV-VP40 using TransIT-LT1 (Mirus) following the manufacturer's instructions, whereas pCAGGS-eGFP-transfected cells served as negative controls. 48 hours post transfection, cells were lysed in 4x SDS-PAGE sample buffer, and 50 µl 4x SDS-PAGE sample buffer was added to 150 µl supernatant. SDS-PAGE and semi-dry Western blotting was performed using an ethanol-containing blotting buffer (5.8 g Tris(hydroxymethyl)aminomethan; 2.9 g Glycine; 200 mL Ethanol; ad 1 mL H_2O) together with nitrocellulose membranes and a blotting current of 0.8 mA/cm^2 for 90 min. A 7% skim milk powder solution in PBS was used for blocking. For detection, a mouse anti-flag antibody (clone M2, Sigma-Aldrich cat. no. F1804; 1:1.000) and a polyclonal anti-mouse IgG (IRDye 800CW) secondary antibody (926-32210, Li-cor; 1:15.000) were used, and blots were visualized using an Odyssey CLx infrared scanner (Li-cor), and quantified using the Li-cor Image Studio Lite v5.2 software.

For the trVLP assay, 293T (p0) cells in 12-well format were transfected with expression plasmids encoding the EBOV RNP proteins, a T7-driven tetracistronic EBOV minigenome containing a Renilla luciferase reporter and encoding either the EBOV or BOMV glycoprotein

gene (8A variant), the T7 RNA polymerase, as well as firefly luciferase for normalization. 72 hours post transfection reporter activity in p0 cells was measured using a Glomax Discover multimode reader (Promega). trVLPs from p0 cells were used to infect 293T target (p1) cells pretransfected with plasmids encoding EBOV RNP proteins as well as TIM-1, and after 72 hours reporter activity in p1 cells was measured.

Rescue, titration and growth kinetics of recombinant virus

For rescue of recombinant BOMV, Huh7 cells (p0 cells) in 6-well plates were transfected with 250 ng of pAmp-rgBOMV as well as plasmids encoding the EBOV RNP proteins (125 ng pCAGGS-NP, 125 ng pCAGGS-VP35, 75 ng pCAGGS-VP30, 1000 ng pCAGGS-L) and T7 polymerase (250 ng pCAGGS-T7opt) using TransIT-LT1 (Mirus) following the manufacturer's recommendations. Medium on the cells was exchanged after 1 day. After 1 week 1 ml of supernatant was passaged onto Vero E6 cells (p1 cells), and these were continuously monitored for the development of CPE. A single medium change was done after 24 days. For stock production, 1 ml of p1 supernatant harvested 31 days after blind passage was used to infect Vero E6 cells in a T175 flask. This virus stock was harvested after 18 days, when CPE became apparent, cleared of cellular debris, and frozen in liquid nitrogen.

Cryo-Electron Tomography of BOMV and VLPs

For structural studies, BOMV was inactivated by 4% paraformaldehyde and 0.1% glutaraldehyde (EM-grade, EMS) prior to removal from BSL4. Solutions containing fixed BOMV or VP40-VLPs were mixed with 10 nm protein A-coated colloidal gold (Aurion) and applied onto glow-discharged electron microscopy grids (200 mesh, R 2/1, Quantifoil) just before vitrification of the samples by plunge-freezing using a Leica EM GP2 automatic plunge freezer.

Data were acquired at Heidelberg University on a Titan Krios Transmission Electron Microscope (ThermoFisher Scientific) operated at 300 keV and equipped with a K3 direct electron detector (Gatan) and a BioQuantum® LS energy filter with a slit width of 20 eV. Maps were acquired at 8700 x magnification (pixel size: 10.64 Å) and tilt-series were acquired at 33,000 x magnification (pixel size: 2.671 Å) using a dose-symmetric acquisition scheme with an electron dose of approximately 3 $e^{-}/Å^{2}$ per projection with tilt ranges from 60° to -60° in 3° increments using SerialEM. Tilt series were reconstructed using the IMOD software package, including alignment of the stacks using gold fiducials, 3D contrast transfer function (CTF) correction and dose-filtration. Final reconstructions were performed by weighted back-

projections with a simultaneous iterative reconstruction technique (SIRT)-like filter equivalent to 10 iterations. For visualization, final tomograms were binned 3 x, 10 slices were averaged and low-pass filtered.

Immunofocus assay

Organs were weighed and homogenized in tubes containing 1 mL DMEM and Lysing Matrix D beads (MP Biomedicals) using a FastPrep-24TM 5G tissue lysis homogenizer. Vero E6 cells seeded at more than 95% of confluency in 24-well plates were incubated with 200 μL of serial 10-fold dilutions of homogenized organs and blood samples. After 1 hour of incubation at 37 °C, the inoculum was removed and replaced with a 1% methylcellulose medium overlay. The overlay was removed after 7 days, and cells were fixed with 4% formaldehyde, washed with water, and permeabilized for 30 minutes with 0.5% Triton X-100 in PBS. After washing with water, cells were blocked with 5% FCS PBS. Cell foci were detected using an anti–Ebola-NP antibody (clone KZ51 IgG1 mouse, Absolute Antibody) at a dilution of 1:2000 at room temperature for 1 hour. For detection of foci, cells were washed with water and incubated with 200 μL containing secondary peroxidase-conjugated AffiniPure Sheep Anti-Mouse IgG (H+L) antibody (515-035-003, Jackson ImmunoResearch Laboratories Inc.) at a dilution of 1:40,000 for 1 hour at room temperature. After washing with water, foci were visualized with tetramethylbenzidine (TMB) and counted.

Flow cytometry

Organs were cut into small fragments and digested for 30 minutes at 37°C with Collagenase D (2 mg/mL) (Roche) and DNase (50 μg/mL) in RPMI-1640 medium. In order to obtain single-cell suspensions, tissue fragments were further disrupted by passage through a 70-μm nylon cell strainer (BD Biosciences). Lysis of red blood cells was done with BD Pharm Lyse buffer (BD Biosciences) following the manufacturer’s instructions. Cells were then stained for viability with Zombie NIR (BioLegend) for 30 minutes at room temperature, washed twice with PBS, and blocked with Human TruStain FcX (Fc receptor blocking solution, BioLegend) for 20 minutes at room temperature. Staining of the cells was done with an antibody cocktail consisting of the following multiparametric flow cytometry panel: CD45-FITC (2D1; 368507, BioLegend), CD1c-APC (L161; 331523, BioLegend), CD38–PerCP-Cy5.5 (HIT2; 303521, BioLegend), CD16-PECy7 (3G8; 302015, BioLegend), CD141-PE (M80; 344103, BioLegend), HLADR-BV785 (L243; 307641, BioLegend), CD8-BV570 (RPA-T8; 301037, BioLegend), CD14-BV510 (M5E2; 301841, BioLegend), CD56 (HCD56; 318325,

BioLegend), CD19-PB (HIB19; 982404 BioLegend), CD4-BUV737 (SK3; 564305, BD Biosciences), and CD3-BV650 (OKT3; 317323, BioLegend). Formaldehyde-inactivated samples were acquired using an LSRFortessa (BD Biosciences) and analysed with the FlowJo software (BD Biosciences).

Histopathology

A full autopsy was performed on all animals under BSL4 conditions. Tissue samples including lung, liver, spleen, kidney, and brain were collected, fixed in 10% neutral-buffered formalin and trimmed for paraffin embedding. The whole lung, one cross section of the liver and spleen, and a sagittal section of the brain and kidney were embedded in paraffin and 2-3 μm-thick sections were stained with hematoxylin and eosin. Consecutive slides were processed for immunohistochemistry (IHC). Briefly, sections were mounted on adhesive glass slides, dewaxed in xylene, followed by rehydration in descending graded alcohols. Endogenous peroxidase was quenched with 3 % hydrogen peroxide in distilled water for 10 minutes at room temperature. Antigen heat retrieval was performed in a steamer for 20 minutes followed by a cooling period. Nonspecific antibody binding was blocked by pure goat normal serum for 30 minutes at room temperature. The primary antibodies were applied overnight at 4°C (anti-Ebola NP, Ab00692-23.0, Absolute Antibody, 1:1000), and the secondary biotinylated goat anti-rabbit antibody was applied for 30 minutes at room temperature (Vector Laboratories, 1:200). Immunolabelling was visualized by 3-amino-9-ethylcarbazole substrate (AEC, Dako, Agilent) producing a red-brown signal and sections were counter-stained with Mayer's hematoxylin. Slides were scanned using a Hamamatsu S60 scanner, and evaluation was performed using the NDPview.2 plus software (Version 2.8.24, Hamamatsu Photonics K.K.).

For all tissues, the distribution of viral antigen was semi-quantitatively scored on an ordinal 0 to 4 scale within the most severely affected high power field (40x, 400 x 800 µm area): 0 = negative, 1 = focal to oligofocal (1- 3 foci, <5% of the tissue affected), 2 = multifocal (>3 foci, 6-40%), 3= coalescing (41-80%), 4 = diffuse (>81%). The target cell was specified based on the location and cellular morphology.

For evaluation of histopathologic changes, specific criteria are given in Suppl. Table S2. Briefly, changes were recorded on ordinal scores using the tiers: 0 = no change, 1 = minimal reflecting focal to oligofocal changes (<5% of the tissue affected), 2 = mild or multifocal (6-40%), 3= moderate or coalescing (41-80%), 4 = severe or diffuse (>80%) changes. The occurrence of viral inclusion bodies and activation of endothelium was recorded as present

(score 1) or absent (score 0). The sum of all values resulted in the tissue specific lesion score per animal.
